# The relationship sabotage scale: an evaluation of factor analyses and constructive validity

**DOI:** 10.1186/s40359-021-00644-0

**Published:** 2021-09-19

**Authors:** Raquel Peel, Nerina Caltabiano

**Affiliations:** 1 School of Psychology and Counselling, University of Southern Queensland, Building B; Room 340A, 11 Salisbury Road, Ipswich, QLD 4305 Australia; 2 Department of Psychology, James Cook University, 1/14-88 McGregor Road, Smithfield, Cairns, QLD 4878 Australia

**Keywords:** Romantic relationships, Relationship sabotage, Self-sabotage, Defensiveness, Trust difficulty, Relationship skills, Scale development, Relationship sabotage scale

## Abstract

**Background:**

Some individuals are no longer entering romantic relationships, others move through relationships too quickly searching for “the one” and making quick assessments of their romantic partners, while others stay in their relationships but “check out” or do not work on their issues. These are conclusions from two studies: (1) an interview with psychologists who specialise in relationship therapy, and (2) an analysis of individuals’ lived experiences of relationships. The concept of relationship sabotage can explain these phenomena. However, presently, there is no instrument to conceptualise and empirically measure how people continue to employ self-defeating attitudes and behaviors in (and out) of relationships to impede success, or withdraw effort, and justify failure.

**Methods and Results:**

A series of three studies (involving a total of 1365 English speaking individuals of diverse gender orientation, sexual orientation, and cultural background, with relationship sabotage experience) were conceptualized for the current project to fill the need for scale development and to build empirical evidence on the topic of self-sabotage in romantic relationships. The scale was developed over two studies using exploratory factor analysis and one-congeneric model analyses. The third study, using confirmatory factor analysis, confirmed the final structure for the Relationship Sabotage Scale (RSS), which contains 12 items and three factors: defensiveness, trust difficulty, and lack of relationship skills. Constructive validity analyses were also conducted.

**Conclusion:**

The RSS is a brief scale that provides conclusive information about individual patterns in relationships. Findings using this scale can offer explanations regarding the reasons that individuals engage in destructive behaviours from one relationship to the next. Investigations should continue to test a model for sabotage in romantic relationships using the developed scale and other factors such as relationship differences and insecure attachment. More specifically, this measure can be used to understand mediator constructs of relational outcomes within the attachment framework to explain relationship dissolution and work towards relationship maintenance.

## Introduction

Up until recently, the term ‘self-sabotage’ had not been used empirically in the context of romantic relationships. The literature discussing self-defeating patterns in intimate relationships [1] suggests relationship sabotage is a product of goal-oriented defensive strategies informed by attachment styles. This premise is highlighted in Rusk and Rothbaum’s work [2, 3], which illustrates how attachment and goal orientation theory can be integrated to explain individuals’ functioning during times of stress in intimate engagements.

### Attachment and goal orientation theory

The idea that attachment is a continuous and persistent process was first encouraged by Bowlby’s [4] statement “from the cradle to the grave”. Following Bowlby’s logic, Hazan and Shaver [5] explored the continuity of attachment styles into adulthood and conducted ground-breaking research pioneering the investigation of romantic love as part of the attachment process. Hazan and Shaver’s [5] research investigated individuals’ relationship experiences and working models. The authors concluded that schemas derived from working models of the self and others in relationships in childhood and adulthood can place insecure individuals in a “vicious cycle”, where previous experiences affect beliefs, leading to predicted outcomes [5].

Just as it is defined in childhood, adult attachment styles are also based on two dimensions: (1) attachment-related anxiety and (2) attachment-related avoidance. Individuals in the first dimension are typically focused on their sense of self-worth as characteristic of their relationship with others (i.e., acceptance vs. rejection). Those who are anxiously attached expect, readily perceive, and overreact to the possibility of being rejected. While individuals in the second dimension typically define their level of comfort in a relationship with others as a function of intimacy and interdependence with others. Those who are avoidant also tend to deny and suppress a desire for romantic engagement [1, 5–7]. Further, Collins et al. [6] proposed that secure individuals hold a positive relational schema with optimistic expectations of others, while insecure individuals hold a vulnerable relational schema that predisposes them to perceive relationships as mostly negative. More specifically, Collins et al. [6] found that, when anxious individuals were faced with hypothetical partner transgressions, they became emotionally distressed, adopted relationship-threatening attributes and held maladaptive behavioural intentions. The same was not found for individuals high in avoidance [6]. Therefore, it is concluded that different forms of insecure styles are linked with distinct patterns of behaviour. Overall, the main differences between anxious and avoidant individuals is the way they understand intimacy, the way they deal with conflict, their attitude towards sex, their communication skills, and their relationship expectations [8]. Taken together, the evidence shows that, compared with secure individuals, insecure individuals are more likely to understand their partner’s behaviour as negative.

The intersection between attachment and goal-orientation theories presents an interesting line of research. While attachment theorists are concerned with how the relationship between infant and caregiver influences socioemotional functioning, most goal-orientation theorists examine how individuals’ views and goals might lead them to a constructive or defensive response to stressful situations [2]. In other words, entity views, activated by insecure attachment styles during times of stress, can foster self-validation goals, leading to defensive strategies to protect self-worth [2]. In accordance, Elliot and Reis [9] suggested that self-sabotage is possibly enacted when individuals are insecurely attached, hold avoidance goals for their relationship, and are driven to self-protect, as oppose to seek proximity. Research conducted by Locke [10] also showed that insecure attachment predicts weaker goals to approach closeness with a romantic partner. Additionally, Kammrath and Dweck [11] found that individuals with insecure attachment often expect their relationships will fail, which in turn means they were less likely to express concerns and engage in strategies to resolve issues with their partners.

These findings show that when individuals do not feel supported, they are unable to learn from stressful situations and continue to develop and grow their relationships. In this case, their instinct is often to self-protect and their goal to form and maintain relationships becomes secondary to managing the risk of potentially hurtful outcomes [12, 13]. Conclusively, defensive strategies can become self-defeating and, in turn, hinder individuals’ chances of a successful relationship. Thus, it is possible that patterns of relationship attitudes and behaviors resulting from individual differences might be contributing to a cycle of relationship sabotage.

Altogether, the literature has long addressed the influence of individuals’ adult attachment styles on the maintenance and dissolution of intimate engagements. Now, some compelling research has been conducted using both attachment and goal-orientation theories towards understanding relationship sabotage. In particular, two studies which have gathered the insight from psychologists specializing in relationship therapy [14] and analyzed individuals’ lived experience in relationships [15], have served as the basis for the current project. Thus, the overall aim of the series of studies described here is to fill the need to conceptualise and empirically measure relationship sabotage.

### Defining relationship sabotage

Self-sabotage is generally explained as a synonym of self-handicapping. However, the practice of self-handicapping is limited mainly to physical barriers employed to explicitly hinder performance driven activities, usually found in the educational and sport contexts. In these contexts, self-handicapping is defined as a cognitive strategy employed with the overall aim of self-protection [16, 17]. More specifically, self-handicapping happens when one creates obstacles which impede success or withdrawal effort in order to protect their self-esteem and competent public and private self-images. Thus, this definition does not fully encompass complex intrinsic behaviors commonly observed in romantic relationships.

Originally, Post [18] proposed that self-sabotage can be used in the organisational context to explain maladaptive behavioural expressions of individuals dealing with intrapersonal struggles. Thus, the term “sabotage” better fits the description of self-defeating attitudes and behaviors that can lead to relationship sabotage. Fusing this definition with that of self-handicapping, the two previous studies conducted by Peel and colleagues [14, 15] offered a novel definition for self-defeating attitudes and behaviors in relationships. Similarly to how self-handicapping is understood, relationship sabotage can be defined as a pattern of self-defeating attitudes and behaviors in (and out) of relationships employed to impede success, or withdraw effort, and justify failure. However, this description is not exhaustive. Individuals who display these attitudes and behaviors also appear to hold insecure views of romantic relationships and, although they might be doing all they can to start and maintain the relationship [19], failure is an expected outcome in the short or long-term future [2, 3].

Although the novel working definition for relationship sabotage has been well accepted by practicing psychologists and the general public, relationship sabotage does not look the same for all individuals. The two previous studies conducted by Peel and colleagues [14, 15] showed different ways in which sabotage is presented in relationships. Some sabotage by not entering relationships. This is due to a belief that they are not worthy or that the relationship is not going to work. Others are stuck in a cycle of successfully initiating a relationship, yet being unable to maintain long-term engagements, and embarking on a path that appears to be a destined break-up. In this case, individuals are moving through relationships too quickly searching for “the one” and making quick assessments of their romantic partners. While others sabotage by staying in their relationships long-term, despite being unsatisfied or unhappy. In this case, individuals have “checked out”, or have lost hope, and are no longer working on their issues, thus hindering their chances of relationship satisfaction.

Altogether, there are three main examples of how sabotage is presented in relationships. Nevertheless, one common theme to explain motivation amongst all these cases is fear. Participants in the Peel and Caltabiano study [15] shared their “heartbreak” stories and explained how fear of being hurt again, fear of rejection, or fear of abandonment prevent them from trying new relationships. Fear was also mentioned as a motive for why individuals avoid committing to relationships. Additionally, participants explained they avoid working on their relationships due to poor self-esteem or self-concept and loss of hope. Overall, it is fear which motivates individuals to engage in defensive strategies. Yet, to be discussed are possible self-defeating attitudes and behaviors which could be classified as symptomatic of relationship sabotage. To this end, the next two section will review themes discussed in the 2019 and 2021 studies conducted by Peel et al. [14, 15].

### Relationship sabotage in the counselling context

The 2019 study [14] investigated how relationship sabotage is presented in the counselling context and understood by practicing psychologists (with over 20 + years of experience), specializing in relationship therapy. This was an inductive qualitative study, conducted prior to defining the phenomenon of relationship sabotage, towards understanding possible accounts for individual motivation and representative self-defeating attitudes and behaviors. Overall, this study has provided preliminary evidence for how to define relationship sabotage and how to identify attitude and behaviors that are symptomatic of relationship sabotage. Psychologists described attitudes and behaviors that are well understood to be maladaptive in romantic relationships in accordance with experts in the field, such as John Gottman and Susan Johnson and colleagues [20–25]. It seems that people sabotage romantic relationships primarily to protect themselves, as a result of insecure attachment styles and past relationship experiences, and the many ways they do this was represented over 12 main themes: (1) partner attack (e.g., criticism and lack of communication skills), (2) partner pursuit (e.g., clinginess), (3) partner withdrawal (e.g., stonewalling), (4) defensiveness, (5) contempt, (6) self-esteem issues, (7) controlling tendency (e.g., controlling partner’s finances), (8) lack of relationship skills, (9) trust difficulty, (10) destructive tendency (e.g., excessive drinking), (11) attitude to affairs, and (12) relationship belief. Interestingly, practitioners interviewed in this study highlighted that the same attitudes and behaviors that are initially employed to make the relationship function well are the contributors to relationship dissolution in the short or long term. Please see Peel et al. [14] for a detailed discussion of this study and the 12 themes discovered through applied thematic analysis.

### Individuals’ Lived Experience of Relationship Sabotage

The themes from the 2019 study [14] were confirmed in a follow-up deductive qualitive study in 2021 [15], which investigated individuals’ lived experiences of relationship sabotage and maintenance over participants’ lifespan. More specifically, participants explained how relationship sabotage happened for them over several relationships. Defensiveness, trust difficulty, and lack of relationship skills were the most salient themes contributing to relationship sabotage. Defensiveness is a self-protection strategy used as a counterattack when feeling victimised against a perceived attack. In support, Gottman [26] explained that defensiveness is often a result of perceived criticism and contempt, and in turn, can trigger a cascade of behaviours leading to relationship dissolution (e.g., stonewalling). Trust difficulty is often a result of past experiences of betrayal. This theme included being unable to trust romantic partners and feeling overly jealous. Lack of relationship skills refers to participants’ inability to understand or have insight into the dynamics involved in a coupled relationship. For instance, lack of experience, inflexibility, immaturity and learned helplessness were categorized under this theme as contributors.

### The case for the relationship sabotage scale

Although the literature discussed thus far is abundant, a major gap in understanding relationship sabotage still exists. Presently, there is no instrument to conceptualise and empirically measure how people continue to employ self-defeating attitudes and behaviors in (and out) of relationships. To this end, the twelve main themes identified by psychologists in the 2019 study [14] have helped inform the generation of the initial item pool. However, not all twelve themes are expected to be confirmed as separate constructs, as some are attitudes and behaviors to explain why individuals sabotage their relationships, while others represent attitudes and behaviors to explain how sabotage happens. Therefore, the results from the 2021 study [15], which highlighted defensiveness, trust difficulty, and lack of relationship skills, have also served to identify the most prominent themes and those most likely to be represented as separate constructs.

### Current Project

A series of three studies were conceptualized for the current project to fill the need for scale development and to build empirical evidence on the topic of sabotage in romantic relationships. The first study was designed to pilot test the list of items using an exploratory factor analysis (EFA). This was an important step as not all 12 themes were expected to be represented as unique and separate constructs in the final scale. The second study aimed to refine the scale and factor structure using a two-part EFA and one-congeneric model analyses. Lastly, a third study examined the final structure for the Relationship Sabotage Scale (RSS) with a confirmatory factor analysis (CFA) and reliability and construct validity analyses.

## Methods and results

The following section will collectively present the methods employed and the results found to best illustrate the sequential steps of scale development, refinement, and validation.

### Procedure

Ethics approval were obtained from the Human Ethics Committee at James Cook University and the University of Southern Queensland (Number H7414; Number H20REA042). The current studies were conducted online as an anonymous survey. The online link was placed on sites, such as the primary and fellow researchers’ website; Facebook and Twitter; and the universities research participation system. Snowball recruitment (i.e., participants sharing the information sheet or web link with other potential participants) was also encouraged. It is estimated that participants took around 15–30 min to complete the survey. Data for the current studies were collected between June 2018 and December 2020 in three separate campaigns. Data were analyzed using SPSS and AMOS (IBM Statistics), version 25.

### Item generation

The initial items pool were generated based on the 12 main themes extracted from the thematic analysis of interviews conducted with psychologists specializing in relationship therapy, reported in the 2019 study [14]. Although items were created based on these broad themes, it was not expected all themes would be represented as separated constructs. Instead, it was expected that constructs would be an agglomeration of the specified themes as per the 2021 study [15]. Additionally, as per Worthington and Whittaker’s [27] recommendation, the newly formulated items were submitted to expert reviewers (KMB; BB) in the field of relationships research. Both reviewers are practicing psychologists with experience in relationship counselling. Feedback from the reviewers resulted in additional items being added (three items were added to the initial pool of 57 items, resulting in a total of 60 items, with an approximately equal number of items per theme) and changing the wording of some items for better comprehension. Finally, reverse questions were included to combat response automatism and a seven-point Likert scale, ranging from 1 (“strongly disagree”) to 7 (“strongly agree”) was used, where high scores indicated high levels of the measured dimensions. The items were randomly presented as a survey to prevent question order from affecting scores. In the survey, participants were instructed with the following message: “The following statements concern how you feel and behave in romantic relationships. We are interested in how you generally experience relationships, not just in what is happening in a current relationship. If you are not in a relationship, think back to your last relationship. Please respond to each statement by indicating how much you agree or disagree with it”. See Table 1 for a complete list of the items included in the survey.

**Table 1:** List of themes and proposed items for the Relationship Sabotage Scale

Themes	Proposed items
Partner attack	1. I often criticise my partner 2. I tend to focus on the things my partner does not do well 3. When I think about my partner, I focus on the things that attracted me in the first place 4. I communicate well with my partner 5. Fights with my partner often end with yelling and name calling
Partner pursue	6. I like to know what my partner is doing when we are not together 7. I understand if my partner does not reply to my text or phone call straight away 8. I get upset about how much time my partner spends with their friends 9. I get anxious when I think about my partner breaking up with me 10. I check-in with my partner after arguments to see if we are still okay 11. I like to check if my partner still loves me
Partner withdraw	12. I sometimes hide my emotions from my partner 13. I prefer to avoid fighting with my partner as I do not like conflict 14. I try not to get too intensely involved in romantic relationships 15. I like to discuss issues in the relationship with my partner 16. Sometimes I feel that distancing myself from the relationship is the best approach 17. Sometimes I spend time with my friends or go online to have a break from the relationship
Defensiveness	18. I get blamed unfairly for issues in my relationship 19. I often feel misunderstood by my partner 20. I have valid reasons for when things go wrong in the relationship 21. I feel like I am unlucky in romantic relationships 22. I feel like I am always being tested in my relationships as to whether or not I am a good partner 23. I constantly feel criticised by my partner
Contempt	24. The way my partner behaves sometimes makes me feel embarrassed 25. I feel like my partner is ashamed of me 26. When I notice that my partner is upset, I try to put myself in their shoes so I can understand where they are coming from 27. I feel respected by my partner 28. My partner makes me feel a lesser person
Self-esteem issues	29. I feel like I always fail at relationships 30. I am the reason why there are issues in my relationships 31. The success of my romantic relationships reflects how I feel about myself 32. I would do a lot better in my relationships if I just tried harder 33. I feel that I am not worthy of my partner
Controlling tendency	34. I like to have control over my partner’s spending 35. I would respect my partner’s decision to leave me if that is what they want 36. I sometimes pretend I am sick to prevent my partner from getting upset with me 37. I believe that to keep my partner safe I need to know where my partner is 38. When it comes to my relationship with my partner I know best
Lack of relationship skills	39. I believe that I do not have to change how I am in relationships 40. I am open to finding solutions and working out issues in the relationship 41. I will admit to my partner if I know I am wrong about something 42. I am open to my partner telling me about things I should do to improve our relationship
Trust difficulty	43. I find it difficult to trust my romantic partners 44. I often get jealous of my partner 45. I sometimes check my partner’s social media profiles 46. I do not always believe when my partner tells me where they have been or who they have been with
Destructive tendency	47. I like to spoil myself more than I should 48. I enjoy partying and I am always looking to have a good time 49. My partner often complains about how much money I spend 50. My partner often complains I drink too much
Attitude to affairs	51. I would forgive my partner if I found out they had an affair 52. I believe having affairs is part of being in a romantic relationship 53. My partner should forgive me if I have affairs 54. If I have an affair it will be because my partner neglects me
Relationship belief	55. If my relationship is not working, I will end it and look for another one 56. I do not waste time in relationships that are not working 57. I believe someday I will have a great romantic relationship with someone 58. I believe that some relationships are doomed from the start 59. I am happy when I feel like my relationship is just meant to be 60. A successful relationship takes hard work and perseverance

Reverse questions—3, 4, 7, 15, 26, 27, 35, 40, 41, 42, 57, 60

### Study participation criteria

Participants for the three studies were English speaking individuals of diverse gender orientation, sexual orientation, and cultural background, with lived experience of relationship sabotage.

### Study 1

#### Sample

A sample of 321 participants was recruited for this study. A sample size above 300 is considered acceptable for EFA [27–30], especially given that the sample item communality values were within the recommended range (0.40–0.90), with few exceptions. Participants’ ages ranged between 15 and 80 years (*M* = 29.60, *SD* = 13.42), where five participants did not disclose their age. The distribution included 98 male participants (30.5%), 222 female participants (69%) and one reported as ‘other’ (0.5%). Regarding sexual orientation, most participants reported being heterosexual (243, 76%), while 53 (17%) self-identified as bisexual, 11 (3%) self-identified as homosexual, 11 (3%) reported as ‘other’, and three (1%) elected not to answer. For those who reported as ‘other’, 11 provided descriptions for their sexuality, which included androphilic (one), asexual (three), asexual and homoromantic (one), asexual and romantic (one), bisexual (one), heteroflexible (one), pansexual (one), polysexual (one) and queer (one). Most participants (193, 60%) reported being in a relationship (i.e., committed, de facto, married), with a reported mean of 7.1 years (*SD* = 10.39, range 0–59) for their longest relationship duration, and a total of 99 (31%) participants reported having had an affair. In addition, a total of 78 (24%) participants reported previously seeing a psychologist or counsellor for issues regarding a romantic relationship. Participants were all English speakers, from the United States (96, 30%), Australia (53, 16.5%), and Other (172, 53.5%).

#### Item analysis

The data for this first study showed mild deviations from normality with skewedness values ranging from − 1.09 to − 1.69 and kurtosis values ranging from − 1.37 to 2.62. This complies with the parameters recommended by Fabrigar et al. [28] to treat the data as normally distributed (i.e., skewness < 2, kurtosis < 7). Lastly, the sample did not include missing data.

#### Analysis of initial item pool and underlying factor structure

The aim of this initial analysis was to assess the original item pool, the underlying factor structure for the proposed inventory, reduce the number of items, and determine the highest loading items. As per Costello and Osborne’s [29] and Carpenter’s [30] recommendation, a maximum likelihood (ML) extraction method was applied when conducting EFA. This extraction method is arguably the most robust choice for normally distributed data, as it provides more generalizable results and allows for the computation of goodness-of-fit measures and the testing of the significance of loadings and correlations between factors [28–31]. These are important considerations [32] for future analysis of the scale using structural equation modelling (SEM). The data factorability was examined with the Kaiser–Meyer–Olkin (KMO) measure of sampling adequacy [33] and Bartlett’s test of sphericity [34]. The KMO statistic measures whether the correlations between pairs of variables can be explained by other variables [33]. The Bartlett’s test measures whether the correlation matrix differs significantly from an identity matrix [34]. These are necessary conditions to support the existence of underlying factor structures.

Factorability was established with a KMO above the recommended (i.e., 0.6) at 0.84 and the Bartlett’s test was significant (*χ*^2^_(1,770)_ = 8004.04, *p* < 0.001). Eigenvalues above 1, as per Kaiser’s [35] recommendation, indicated 15 factors, accounting for 62.49% of the total variance in the test. Factor 1, the strongest factor, accounted for 17.65% of the variance. All the remaining factors explained less than 10% of the total variance. Overall, the factor correlation matrix showed that factors were not highly correlated (i.e., < 0.3), which indicated the existence of unique factors. An inspection of the screeplot revealed a break after the sixth component. Next, a parallel analysis was conducted, and results showed eight components with eigenvalues exceeding the corresponding criterion values for a randomly generated data matrix of the same size (60 variables × 321 respondents). To ensure a conservative approach at this stage, eight components were retained for further investigation.

The eight-component solution explained a total of 48.68% of the total variance, with eigenvalues of 10.6, 4.5, 3.5, 2.9, 2.4, 2.1, 1.8, and 1.7, respectively. To aid in the interpretation of results, a direct oblimin rotation with Kaiser normalization was performed, which allowed for factors to correlate. It was assumed that factors within the construct of relationship sabotage should all correlate [30], as this is often the case when measuring psychological constructs [28, 29]. The pattern and structure matrices were reviewed, and the rotated solution showed all components included moderate to strong loadings (i.e., between 0.32 and 0.89), with the majority of items loading substantially on only one component. Further investigation to ensure the quality of items was also applied. Items loading with coefficient values below 0.3, or loading on more than one factor with coefficient values above 0.3, were removed [27, 29, 30, 36]. This resulted in 19 items dropped, with a total of 41 items remaining.

### Study 2

#### Sample

A sample of 608 participants was recruited for this study. This sample size was deemed appropriate based on specific recommendations. Bentler and Chou [37], Worthington and Whittaker [27], and Kline [32] recommended a sample of a minimum of 200 participants and a minimum of 5:1 participants per parameter. In the current study, the most complex model estimated 16 parameters (a ratio of 38:1). Therefore, the current sample was adequately powered to detect significant misspecifications in the models examined. Further, Browne [38] developed the Asymptotic Distribution Free (ADF) estimator for sample sizes based on a weight matrix in the function for fitting covariance structures. This method is considered too stringent [39] and other methods, such as the aforementioned, are most often used. Nevertheless, it is noted that the current study met the sample size suggested by the ADF estimator, with 608 participants for 8 observable variables and 1 latent variable in the most complex model.

Participants’ ages ranged between 17 and 80 years (*M* = 32.30, *SD* = 13.76) and five participants did not disclose their age. The distribution included 156 male participants (26%) and 452 female participants (74%). Regarding sexual orientation, the majority of participants reported being heterosexual (486, 80%), while 77 (12.5%) self-identified as bisexual, 28 (4.5%) self-identified as homosexual, 12 (2%) reported as ‘other’, and five (1%) elected not to answer. Most participants (394, 65%) reported being in a relationship (i.e., committed, de facto, married), with a reported mean of 8.6 years (*SD* = 10.36, range 0–61) for their longest relationship duration, and a total of 183 (30%) participants reported having had an affair. In addition, a total of 210 (34.5%) participants reported previously seeing a psychologist or counsellor for issues regarding a romantic relationship. Participants were all English speakers, from the United States (86, 14%), Australia (346, 57%), and Other (176, 29%).

#### Item analysis

The data set for this study showed mild deviations (skewness < 2, kurtosis < 7) and was treated as normal. The sample did not include missing data.

#### Final scale refinement

A two-part EFA was conducted. The first part was the scale refinement process (including factor and scale-length optimization). The second part, recommended by Henson and Roberts [40] and Worthington and Whittaker [27], was to ensure that factor and item elimination does not result in significant changes to the instrument.

The 41 items derived from the previous study were tested for the first part of Study 2. Factorability was established with a KMO at 0.87 and the Bartlett’s test [34] was significant (*χ*^2^_(820)_ = 7,465.817, *p* < 0.001). Eigenvalues indicated eleven factors over 1. These factors explained 58.36% of the variance. An inspection of the screeplot revealed a break after the second component and the results of a parallel analysis showed seven components with eigenvalues exceeding the corresponding criterion values for a randomly generated data matrix of the same size (41 variables × 608 respondents). Using the results from the parallel analysis, seven components were retained for further investigation.

To ensure a stringent approach to retaining factors and items the following five criteria were applied: (1) item coefficient values ≥ 0.32 (this is to ensure the item total variance equals the minimum recommended 10%), (2) inter-item correlation within factors ≥ 0.3, (3) factor reliability ≥ 0.6, (4) inter-factor correlation ≤ 0.3, and (5) number of items on each factor ≥ 4 [29, 30, 32, 36, 41, 42]. Overall, this approach is to ensure constructs can be represented, ensure good model identification [43], and avoid an inadmissible solution [32] prior to conducting one-congeneric model analyses (the next step). This resulted in six items dropped due to low coefficient values, three items dropped due to low inter-item correlation values, and four factors dropped due to insufficient number of items and low factor reliability, with a total of three factors and 20 items remaining.

As per Holmes-Smith and Rowe’s [42] recommendation, one-congeneric model analyses were fitted for each individual factor to clean each construct and ensure model fit prior to establishing the final list of items. All latent variables were scaled from 1 to 7 (from “strongly disagree” to “strongly agree”) by randomly fixing the factor loading from one of the observable variables (also called the reference variable) from each set of constructs to the value of 1. This process was used to identify and scale the model [44]. Also, alternative marker variables were examined as a means of checking for the robustness of the final models. No items were allowed to covary within constructs. The error terms (associated with observable and latent variables) were also set to the value of 1 and measurement error was assumed to be uncorrelated between items [44].

The *t*-rule method [43] was used to assess model identification. Model identification is assumed if the number of parameters to be estimated in a model does not exceed the number of unique variances and covariances in the sample variance–covariance matrix (calculated using *k*). The most complex model analyzed in this study (Factor 1) had 16 free parameters and 8 observable variables; therefore, it met the *t*-rule requirement (i.e., 16 ≤ 36). Free parameters in the model were also estimated using the ML procedure. In SEM, this practice is recommended by several researchers—e.g., Kline [32]—following the original seminal work of Jöreskog [45]. The ML approach is robust for normal, or near normal data, as it provides close estimates of measurement error and a chi-square distribution closely related to the population of estimation.

In this step, factor score regression weights, variance explained, and measurement error were used to assess the quality of items. Modifications were only applied to improve the model when existing literature, previous research findings, and the results from the current set of studies supported the proposed alterations. Six measures were used to assess model fit: (1) chi-square, (2) root mean square error of approximation (RMSEA), (3) goodness-of-fit index (GFI), (4) comparative fit index (CFI), (5) Tucker-Lewis index (TLI), and (6) standardized root mean square residual (SRMR). Overall, the one-congeneric model approach allows for factors of different weights within the same construct to contribute uniquely and does not assume that items are parallel (i.e., all variables carry the same weight).

*Factor 1* The initial analysis for this factor, containing eight items (16, 18, 19, 22, 23, 24, 27, 28), showed a poor fit (*χ*^2^_(20)_ = 98.824, *p* < 0.001; RMSEA = 0.081 [0.065, 0.097], *p* = 0.001; GFI = 0.959; CFI = 0.969; TLI = 0.957; SRMR = 0.031). Model specifications analysis showed high covariance associated with four items (16, 22, 24, 27). Therefore, these items were removed. The final one-congeneric model with four items (18, 19, 23, 28) showed an excellent fit (*χ*^2^_(2)_ = 4.632, *p* = 0.099; RMSEA = 0.047 [0.000, 0.104], *p* = 0.445; GFI = 0.996; CFI = 0.998; TLI = 0.994; SRMR = 0.010). Altogether, this factor contains three items from the original defensiveness theme (items 18, 19, and 23) and one item from the original contempt theme (item 28).

*Factor 2.* The initial analysis for this factor, containing seven items (6, 8, 9, 37, 38, 44, 45), showed a poor fit (*χ*^*2*^_(14)_ = 47.721, *p* < 0.001; RMSEA = 0.063 [0.044, 0.083], *p* = 0.124; GFI = 0.978; CFI = 0.955; TLI = 0.933; SRMR = 0.037). Model specifications analysis showed high covariance associated with three items (6, 9, 38). Therefore, these items were removed. The final one-congeneric model with four items (8, 37, 44, 45) showed an excellent fit (*χ*^2^_(2)_ = 3.724, *p* = 0.155; RMSEA = 0.038 [0.000, 0.097], *p* = 0.540; GFI = 0.997; CFI = 0.996; TLI = 0.988; SRMR = 0.016). Altogether, this factor contains two items from the original trust difficulty theme (items 44 and 45), one item from the original partner pursue theme (item 8), and one item from the original controlling tendency theme (item 37).

*Factor 3.* The initial analysis for this factor, containing five items (26, 40, 41, 42, 60), showed an excellent fit (*χ*^2^_(5)_ = 7.638, *p* = 0.177; RMSEA = 0.029 [0.000, 0.069], *p* = 0.767; GFI = 0.995; CFI = 0.993; TLI = 0.986; SRMR = 0.021). However, item 60 showed a weak regression weight (i.e., < 0.32) and therefore was dropped. The final one-congeneric model with four items (26, 40, 41, 42) also showed an excellent fit (*χ*^2^_(2)_ = 3.873, *p* = 0.144; RMSEA = 0.039 [0.000, 0.098], *p* = 0.524; GFI = 0.997; CFI = 0.995; TLI = 0.984; SRMR = 0.017). Altogether, this factor contains three items from the original lack of relationship skills theme (items 40, 41, and 42) and one item from the original contempt theme (item 26).

These analyses resulted in eight items dropped. The final EFA was performed on 12 items. Factorability was established with a KMO at 0.84 and the Bartlett’s test [34] was significant (*χ*^2^_(66)_ = 2,315.468, *p* < 0.001). The three-component solution explained a total of 60.3% of the total variance, with eigenvalues of 4, 1.7, and 1.5, respectively. No other factor showed eigenvalues above 1. The rotated solution showed all components included moderate to strong loadings (i.e., between 0.54 and 0.88) and the majority of items loaded substantially on only one component. Factor 1 (33.3%) was termed Defensiveness, Factor 2 (14.3%) was termed Trust Difficulty, and Factor 3 (12.7%) was termed Lack of Relationship Skills. Overall, this result demonstrated the three-factor model is superior to the eight and seven factor solution previously identified. The final inventory of 12 items and their respective loadings can be viewed in Table 2.

**Table 2:** Scale pattern and structure matrix with maximum likelihood extraction and oblimin rotation

Items (*N* = 12)	1		2		3		h^2^
	Pattern matrix	Structure matrix	Pattern Matrix	Structure Matrix	Pattern Matrix	Structure Matrix	
1. I get blamed unfairly for issues in my relationship [18]	**0.754**	**0.782**	0.056	0.359	0.015	0.317	0.614
2. I often feel misunderstood by my partner [19]	**0.715**	**0.757**	0.080	0.370	0.027	0.319	0.579
3. I constantly feel criticised by my partner [23]	**0.881**	**0.877**	− 0.030	0.325	0.021	0.351	.0770
4. My partner makes me feel a lesser person [29]	**0.841**	**0.820**	− 0.041	0.290	− 0.013	0.299	0.674
5. I get upset about how much time my partner spends with their friends [8]	0.102	0.360	**0.599**	**0.652**	0.052	0.232	0.439
6. I believe that to keep my partner safe I need to know where my partner is [35]	− 0.093	0.195	**0.654**	**0.634**	0.073	0.192	0.411
7. I often get jealous of my partner [36]	0.087	0.332	**0.642**	**0.670**	− 0.025	0.159	0.455
8. I sometimes check my partner’s social media profiles [43]	0.010	0.202	**0.541**	**0.531**	− 0.060	0.072	0.285
9. When I notice that my partner is upset, I try to put myself in their shoes so I can understand where they are coming from. **R** [26]	0.016	0.237	0.037	0.170	**0.540**	**0.555**	0.310
10. I am open to finding solutions and working out issues in the relationship. **R** [39]	0.070	0.300	− 0.016	0.158	**0.616**	**0.640**	0.413
11. I will admit to my partner if I know I am wrong about something. **R** [40]	− 0.055	0.186	0.090	0.194	**0.536**	**0.536**	0.295
12. I am open to my partner telling me about things I should do to improve our relationship. **R** [42]	0.022	0.227	− 0.110	0.052	**0.651**	**0.633**	0.411
Eigenvalues	4		1.7		1.5		
Trace	3.2		2.1		1.9		
% Variance	33.3		14.3		12.7		60.3

Coefficients greater than 0.4 are in bold. Final traces are the transformed eigenvalue variance accounted for statistic after rotation. In brackets are the original item number from the 60-item list. Items 1–4 represent Defensiveness, Items 5–8 represent Trust Difficulty, Items 9–12 represent Lack of Relationship Skills. Reverse items are marked with a bold ‘R’. *N* = 608

### Study 3

#### Sample

A sample of 436 participants were recruited for this study. The same specifications to access the appropriateness of sample size as Study 2 were used. Participants’ ages ranged between 14 and 75 years (*M* = 27.41, *SD* = 12.37). The distribution included 128 male participants (29.5%) and 302 female participants (69.5%), and six reported as ‘other’ (1%). For those who reported as ‘other’, six provided descriptions for their gender, which included gender fluid (one), gender neutral (one), non-binary (one), queer (two), and transgender male (one). Regarding sexual orientation, most participants reported being heterosexual (336, 77%), while 74 (17%) self-identified as bisexual, 11 (2.5%) self-identified as homosexual, eight (2%) reported as ‘other’, and seven (1.5%) elected not to answer. For those who reported as ‘other’, eight provided descriptions for their sexuality, which included asexual (two), bi-curious (one), confused (one), panromantic and demisexual (one), pansexual (one), and questioning (two). Most participants (250, 57%) reported being in a relationship (i.e., committed, de facto, married), with a reported mean of 5.68 years (*SD* = 8.13, range 0–50) for their longest relationship duration, and a total of 93 (21%) participants reported having had an affair. In addition, a total of 101 (23%) participants reported previously seeing a psychologist or counsellor for issues regarding a romantic relationship. Participants were all English speakers from the United States (70, 16%), Australia (215, 49%), and Other (151, 35%).

#### Item analysis

The data set for this study showed mild deviations (skewness < 2, kurtosis < 7) and was treated as normal. Also, the sample did not include missing data.

#### Confirmatory factor analysis

A full multi-factor CFA was conducted with the final set of items and the same sample and specifications as the one-congeneric model analyses. The aim of conducting this CFA was to evaluate the EFA-informed factor structure and psychometric properties and to test the fit of the global model. The three factors were represented in the full model by latent variables (fitted as a second-order g model), with each item loading on its respective latent factor, as predicted by the EFA. Factor loadings from one of the observable variables from each set of constructs was randomly set to the value of 1. Also, alternative marker variables were examined as a means of checking for the robustness of the final model. Items were not allowed to load on multiple factors. The three factors were allowed to covary and measurement error was assumed to be uncorrelated between items.

All factors and items significantly loaded in their respective latent factor. Items loaded with *t* values between 6 and 17.2 and regression weights between 0.4 and 0.85. Also, items squared multiple correlations ranged between 0.16 and 0.72. Overall, this indicates items were strong and reliable indicators of the latent variables [44]. The goodness-of-fit statistics demonstrated that the three-factor model had a RMSEA of 0.048 ([0.034, 0.062], *p* = 0.565), which is considered an excellent fit [44]. Although the chi-square value was significant (*χ*^2^_(50)_ = 100.577, *p* < 0.001), this fit statistic is less important than the RMSEA, when fitting a full and more complex model [44, 46]. The RMSEA takes into account the error of approximation in the population and reduces the stringent requirement on the chi-square that the model should hold exactly in the population [44, 46]. An issue with the chi-square statistic is that the more complex the model, the bigger the value and the more likely it is that the model will be rejected. Therefore, the normed chi-squared (*χ*^2^/*df*) was calculated with a value of 2, which is acceptable. The normed chi-square takes model complexity into account and can also be referred to as an index of model parsimony [47].

Regarding incremental or comparative fit indices, the GFI and CFI values were 0.96, which is above the acceptable level. This indicates the hypothesised model accounts for variance in the data well in comparison with the null model. The TLI was 0.95, which is acceptable. This indicates the model is parsimonious. Finally, the SRMR, which is a residual statistic that assesses the residual variance unexplained by the model, showed a level of 0.052, which is also acceptable [48, 49]. Overall, the final 12-item inventory was supported by the CFA.

#### Final scale reliability analysis

Reliability was calculated with the measure of Cronbach’s alpha [50] and the SEM-recommended practice of coefficient *H* [51]. According to Hancock and Mueller [51], coefficient *H* provides a more robust way to assess latent measures created from observable construct indicators, such as regression coefficients, especially if items are not parallel. The Cronbach’s alpha calculation assumes that all items are parallel, which is often not the case, and is affected by the sign of the indicators’ loading. Alternatively, coefficient *H* is not limited by the strength and sign of items and draws information from all indicators (even from weaker variables) to reflect the construct. Further, Lord and Novick [52] proposed that if measures associated with a latent trait are congeneric, which is the case with the current measure, Cronbach’s alpha will be a lower-bound estimate of the true reliability.

The standard cut-off indicators recommended by the most stringent researchers [50, 53, 54] were followed for both analyses (i.e., α ≥ 0.9 = excellent; 0.9 > α ≥ 0.8 = good; 0.8 > α ≥ 0.7 = acceptable; 0.7 > α ≥ 0.6 = questionable; 0.6 > α ≥ 0.5 = poor; 0.5 > α = not acceptable). The results showed acceptable/good reliability for the total scale (α = 0.77; *H* = 0.82), good reliability for Factor 1 (α = 0.85; *H* = 0.87), questionable reliability for Factor 2 (α = 0.60; *H* = 0.62), and acceptable reliability for Factor 3 (α = 0.75; *H* = 0.77). As all sub-scales contain less than ten items, which can affect the reliability value, the mean inter-item correlation value was also inspected. The mean inter-item correlation value for all sub-factors showed a strong relationship between items (i.e., ≥ 0.3).

#### Scale construct validity

Traditional approaches to assess construct validity (i.e., the multi-trait–multi-method [MTMM] matrix approach) rely on the assumption that the construct’s variables are parallel. Therefore, assessing validity with a correlation matrix alone is limited and does not account for the effect of variables with different regression weights and measurement errors. To remedy this limitation, SEM-based approaches to construct validity were also performed. SEM-based approaches highlight how constructs are affected differently and allows them to correlate freely among themselves. Further, these approaches assess how well each construct fits within the model with regards to variance explained and measurement error [55].

*Convergent and Discriminant Validity (MTMM Matrix Approach).* Convergent and discriminant validity were assessed using the MTMM matrix, which assesses construct validity by comparing the correlation matrix between the proposed constructs and constructs measured by different scales, which are either conceptually similar or dissimilar [56]. The three factors were compared with three measures—the Experiences in Close Relationships Scale Short-Form (ECR-SF) [57], used to assess adult insecure attachment styles (i.e., anxious and avoidant attachment); the Perceived Relationship Quality Components Inventory Short-Form (PRQCI-SF) [58], used to assess perceived relationship quality with six components: (1) satisfaction, (2) commitment, (3) intimacy, (4) trust, (5) passion, and (6) love; and the Self-Handicapping Scale Short Form (SHS-SF) [59], used to assess self-handicapping in the educational and sport contexts with mainly physical barriers employed to explicitly hinder performance driven activities. The ECR-SF and PRQCI-SF were used to assess convergent validity and the SHS-SF was used to assess divergent validity.

The sub-factors for the ECR-SF, PRQCI-SF, and SHS-SF were created as per the scales’ manuals, by adding raw scores. The three factors for the scale in development were created as composite variables, which involves using the factor score regression weights obtained from the one-factor congeneric measurement models fitted as part of the CFA, as recommended by Jöreskog and Sörbom [60]. This approach is unlike adding raw scores to represent subscales, which assumes that the items are parallel. Weighted composite variables best represent each variable’s unique contribution. Further, weighted composite variables are continuous, as opposed to Likert scale scores, which are ordinal. Therefore, for the purpose of creating weighted composite variables, factor score regression weights were rescaled to add up to a total of 1.

Regarding convergent validity, Factor 1 (Defensiveness) showed significant positive correlations (*p* < 0.01) with anxious attachment (*r* = 0.348) and avoidant attachment (*r* = 0.435), and significant negative correlation with perceived relationship quality (*r* = ˗0.371). Factor 2 (Trust Difficulty) showed significant positive correlations (*p* < 0.01) with anxious attachment (*r* = 0.508) and avoidant attachment (*r* = 0.197). Factor 3 (Lack of Relationship Skills) showed significant positive correlations (*p* < 0.01) with avoidant attachment (*r* = 0.473) and significant negative correlation with perceived relationship quality (*r* = ˗0.406). Regarding divergent validity, all three factors showed a near zero positive relationship with self-handicapping (ranging between 0.033 and 0.082). See Table 3 below.

**Table 3:** Correlation matrix to measure construct validity

	M (*SD*, range)	D	TD	LRS	PRQ	ANX	AVO	SH
D	2.88 (*1.43*, 1–7)	1	.362**	.263**	− .371**	.348**	.435**	.059
TD	2.91 (*1.15*, 1–6.5)		1	.177**	**− **.082	.508**	.197**	.033
LRS	2.04 (*.78*, 1–7)			1	− .406**	.045	.473**	.082
PRQ	22.99 (*5.69*, 6–30)				1	− .162**	− .567**	**− **.049
ANX	23.58 (*6.86*, 6–41)					1	.279**	.033
AVO	16.11 (*6.44*, 6–35)						1	.058
SH	36.92 (*9.66*, 10–60)							1

** = .01 (two-tailed). Significant coefficients are in bold. RSS and subscales (*N*) = 436, ECR-SF and subscales (*N*) = 436, SHS (*N*) = 436, PRQCI (*N*) = 436. D = Defensiveness, TD = Trust Difficulty, LRS = Lack of Relationship Skills, PRQ = Perceived Relationship Quality, ANX = Anxious Attachment, AVO = Avoidant Attachment, SH = Self-Handicapping

*Convergent Validity (SEM–based Approaches).* According to Bagozzi et al. [55], if all item loadings are statistically significant, meaning that the relationship between an observed variable and latent construct is different to zero, convergent validity can be assumed. Further, Holmes-Smith and Rowe [42] recommended a threshold value of 0.5 for the standardized loading (with a significant *t-*statistic) to achieve convergent validity. Standardized item loadings were in between 0.4 and 0.87 (with a significant *t-*statistic), with all items above 0.5, except for item 37 (0.43), and item 45 (0.4). Additionally, Hair [61] proposed an all-encompassing and more stringent set of criteria for convergent validity, which requires that in addition to standardized factor loading of all items greater than 0.5, an average variance extracted (AVE) between constructs is greater than 0.5, and construct’s composite reliability (CR) is greater than 0.7. This set of criteria is in agreement with Fornell and Larcker’s [62, 63] works. All AVE between factor were above 0.5, with a range of 0.72–1. Further, all factor CR were above 0.7, expect for Factor 2 (0.61), with a range of 0.61–0.84. These results fully supported convergent validity for Factors 1 and Factor 3 and partially support convergent validity for Factor 2. See the Table 4 below for AVE and CR estimates.

**Table 4:** AVE and CR estimates for the relationship sabotage scale factors

Factors	Defensiveness	Trust difficulty	Lack of relationship skill
CR	0.842	0.614	0.752

Pair of factors	Defensiveness and trust difficulty	Defensiveness and lack of relationship skills	Trust difficulty and lack of relationship skills
AVE	0.861	1.01	0.721
Square-rooted AVE	0.928	1.004	0.849
Factors inter-correlation	0.362	0.263	0.177
Squared factors inter-correlation	0.131	0.069	0.031

*Discriminant Validity (SEM–based Approaches).* The criterion adopted by Kline [32] was considered for discriminant validity analyses, which stipulates that validity can be assumed if the correlation between two factors is less than 0.85. This was further supported by Cheung and Wang [64], who recommended the correlation not be significantly greater than 0.7. However, this approach is often criticized for its reliance on the correlation matrix approach, which does not consider variance explained and error measurement [55]. Therefore, two additional approaches were considered.

Discriminant validity was first assessed using the Fornell and Larcker’s [62, 63] approach in a multi-trait–mono-method context using the AVE and inter-correlation between factors. This method showed that all pairs of constructs were distinct, thereby supporting discriminant validity (i.e., AVE > squared factors inter-correlation or square-rooted AVE > factors inter-correlation—refer back to Table 4). Further, discriminant validity was assessed using the Bagozzi et al. [55] nested model method. This procedure involves measuring the difference between the constrained and unconstrained models (with correlations between constructs set to 1) between each two pairs of variables. The conclusion is based on the difference between the models’ chi-square test. The difference between models should show that constraining the correlation between the two constructs worsens the model fit (i.e., there is a significant difference between models), which in turn means that the constructs are discriminant. The nested model approach was performed between factors showing divergent constructs. This confirms there are three distinct factors. Additionally, this approach has gained favor as a technique to compare alternative models [27]. The results from this test fully supported discriminant validity—see Table 5.

**Table 5:** Nested model approach to discriminant validity in the relationship sabotage scale

Models	Defensiveness and trust difficulty			Defensiveness and lack of relationship skills			Trust difficulty and lack of relationship skills		
	*χ*^2^	*df*	*p*	*χ*^2^	*df*	*p*	*χ*^2^	*df*	*p*
Constrained	47.474	19	< 0.000	113.695	19	< 0.000	144.566	20	< 0.000
Unconstrained	36.719	18	0.006	32.675	18	0.018	47.078	19	< 0.000
Difference	10.755	1	0.001	81.02	1	< 0.000	97.488	1	< 0.000

## Discussion

The scale in development, the RSS, underwent an initial EFA in Study 1, a two-part EFA and one-congeneric model analyses in Study 2, and a CFA and construct validity analyses in Study 3. As predicted, not all themes derived from the 2019 study [14], as shown on Table 1, were represented as unique factors in the final scale. Instead, the three themes from the 2021 study [15] study—i.e., defensiveness, trust difficulty, and lack of relationship skills—were represented as distinct constructs. Nevertheless, some concepts were represented as minor sub-themes within the identified constructs in the final measure. For instances, two items from the contempt theme (item 26 and 28) were represented in the defensiveness and lack of relationships skills factors. Another example is the one item from the partner pursue theme (item 8), which was represented in the trust difficulty factor. These findings are all a part of the process of scale development, which although based on a strong literary background, needs to undergo exploratory tests to strengthen the original predictions [30]. Overall, the final scale shows promising psychometrics properties with room for continuing improvement. Following, is a discussion of the three distinct constructs established, the scale’s reliability and construct validity analyses, limitations and future directions, and clinical and theoretical implications.

### Defensiveness

Defensiveness was the strongest factor represented in both the EFA (Study 1 and Study 2) and CFA (Study 3) and this finding was unsurprising. Accordingly, the previous interview study with practicing psychologists revealed that the main reason that people sabotage their relationships is to protect themselves [14]. The same was found when reviewing the accounts of members of the general public with lived experience of relationship sabotage [15]. Further, extensive research [7, 12, 13, 65–67] shows that motivation to self-protect is a powerful reinforcer of maladaptive attitudes and behaviors in relationships with others. Also, De Castella et al. [68] showed that motivation to self-protect goes beyond cultural differences. For instance, in a study comparing Australian and Japanese students regarding academic motivation, the results indicated that self-protectors are typically high in defensive pessimism and self-handicapping, and low in helplessness. This is possibly the same in the context of romantic relationships. Overall, it is well established that adult relationship interactions are strongly guided by a specific set of goals linked to attachment [68], meaning that secure attachment would possibly encourage goals of connection and insecure attachment would encourage goals of self-protection.

The theme of defensiveness encompasses a multitude of attitudes and behaviors. For instance, although three of the final items were from the originally proposed theme, one item belonged to the originally proposed contempt theme (item 28). Defensiveness and contempt items included in the initial item list, similarly to what was proposed by Greenberg and Johnson [23] and Gottman and Silver [69], describe three patterns of communication in the relationship (i.e., attack–attack, attack–withdraw, and withdraw–withdraw). To explain, attacking is understood as a desperate attempt to gain the partner’s attention at any cost. Further, Gottman and Levenson [22] found conflict (expressed as anger, dysfunctional communication, and negativity) to be a strong predictor of marital dissolution. Finally, defensiveness and contempt are two of the “four horsemen of the apocalypse”, described by Gottman and Silver [69] as a clear sign of “marriage meltdown”. Together, these are well-known predictors of relationship dissolution. Therefore, it is understandable that they would amalgamate into one factor in the final scale.

Further, individuals are not likely to resort to the same techniques when self-sabotaging. Therefore, it was expected that not all themes would make a significant contribution. Nevertheless, defensiveness seems to be the one common approach used by people when sabotaging relationships. This result is in accordance with Gottman and Silver’s research [69], which explain defensiveness is a long-term consequence of criticism and contempt. Additionally, people will likely be defensive and engage in their “preferred” destructive technique (e.g., attack or withdraw). Also, individuals who are feeling defensive will often become hyper-vigilant [69], and typically either attack or withdraw [23]. Additionally, Gottman [70] found that 85% of males will resort to stonewalling, which is a known withdrawal approach. In contrast, females are typically known for raising issues in the relationship [70]. Overall, it is agreed that defensiveness is an all-encompassing construct that can take many forms.

### Trust difficulty

Trust difficulty was also significantly represented. Two items from this construct were derived from the originally proposed theme in the initial item pool, with one from the partner pursue theme (item 8) and one from the controlling tendency theme (item 37). There is strong evidence that people who resort to partner pursuit and controlling tendencies, specifically clinginess, will often push their partner away and consequently destroy relationships [19]. Further, there is a strong link between trust difficulty and insecure attachment [5, 71]. Overall, lack of trust is commonly associated with a previous experience of betrayal or the expectation of betrayal [2, 5, 72]. Specifically, Rempel et al. [73] defined trust as a multidimensional trait consisting of three sub-factors (predictability, dependability, and faith), all of which are affected by insecure attachment [74]. Altogether, this construct represents a maladaptive cognition (e.g., mistrust), an emotion reaction (e.g., anxiety), and the resultant behavior (e.g., partner pursuit and controlling tendencies). In accordance, a meta-analysis conducted by Le et al. [75] identified that insecure attachment styles and relationship factors—such as relationship dissatisfaction, lack of commitment, conflict, and trust issues—significantly contribute to the dissolution of a romantic relationship.

### Lack of relationship skills

The practicing psychologists interviewed in the 2019 study [14] proposed that lack of relationship skills is one of the main reasons why people maintain the cycle of relationship sabotage across their intimate engagements. Thus, it was suggested that clients often know little about how relationships works (i.e., what to expect and how to maintain them), which may be a result of poor relationship role models based on negative interactions and outcomes [5, 7, 76]. Consequently, this factor showcased a combination of items; three from the original theme in the initial item pool and one from the original contempt theme (item 26), describing not being able to understand where the other person is coming from, not gathering insight on relationship dynamics, not being open to discuss and work on relationship issues, and not having or displaying problem solving skills. Overall, relationship skills is a broad concept. Therefore, it is likely that it would encompass an amalgamation of concepts. Specifically, partner withdrawal and pursuit (or attack) are well-documented patterns of relationship interaction seen in couples having difficulties communicating [23]. This is further complicated by disrespect, which is a strong characteristic of contempt [69]. Also, individuals with a poor understanding of romantic engagements, often based on unrealistic representations (e.g., fairy tale beliefs), tend to withdraw effort to repair the relationship and giveup easily [77].

### Scale reliability and construct validity analyses

Reliability analyses for the scale in development, conducted in Study 3, showed overall acceptable/good reliability, good reliability for Factor 1, questionable reliability for Factor 2, and acceptable reliability for Factor 3. As expected, Cronbach’s alpha showed a lower-bound estimate of the scale reliability and this is possibly due to the fact that this measure assumes that all items are parallel, which is not the case, and is affected by the sign of the indicators’ loading [47]. In contrast, Coefficient *H* mostly provided stronger estimations, as this measure is not limited by the strength and sign of items and draws information from all indicators (even from weaker variables) to reflect the construct [51, 78]. Nevertheless, Factor 2 showed questionable reliability across both measures, which means this construct needs to be further investigated in different samples and contexts. Also, it is important to note that all sub-scales contain less than ten items, which in turn could have affected the reliability value. For this reason, the mean inter-item correlation value was also inspected, showing a strong relationship between items.

Construct validity was also assessed in Study 3. The first analysis, using correlation matrices, showed convergent validity between the three relationship sabotage constructs, insecure attachment, and perceived relationship quality, as expected. Discriminant validity was established with near zero correlations between the three constructs and self-handicapping. This result is unsurprising. However, the limitations with the MTMM approach, which relies on the assumption that the construct’s variables are parallel, need to be considered. Another issue with using this approach to assess discriminant validity is the fact that most psychological constructs are somewhat related by nature [28, 29]. Therefore, SEM-based approaches were also applied to access construct validity. All the SEM-based methods are considered rigorous and widely accepted. However, there is great debate regarding which practice to use, as no method is without limitations. Cheung and Wang [64] compared approaches using a correlation matrix and SEM for convergent and discriminant validity. As a conclusion, the authors criticized all methods for not having a criterion to effectively address overestimated measurement errors (often as a consequence of using the ML estimation approach) and its influence on translating sample data to population conclusions. Overall, Cheung and Wang [64] recommended that the best approach is to draw conclusions based on a combination of criteria. Specifically, convergent validity can be assumed if the AVE is not significantly less than 0.5 and standardized factor loadings of all items are not significantly less than 0.5, and discriminant validity can be assumed if the correlation between two constructs is not significantly greater than 0.7. Therefore, although the trust difficulty factor’s CR was not above 0.7 (0.614), this would still be considered an acceptable construct as per Cheung and Wang’s [64] recommendation. Further, Holmes-Smith and Rowe [42] proposed that one-factor congeneric models show that all the variables contributing to the overall measurement of the latent variable are similar in nature, meaning that they represent similar “true scores”. As such, a good-fitting one-factor congeneric model implies the construct validity of the construct.

### Limitations and future directions

Future research is needed to continue to improve the psychometrics of the overall relationship sabotage construct. Specifically, items representing trust difficulty and lack of relationship skills might need to be revised and improved. Altogether, the scale will need to be tested in different contexts and with different samples to re-assess reliability and validity. For instance, future studies might involve having relationship practitioners use the instrument with clients. This would be a way to test the measurement in a clinical sample and close the cycle by presenting practitioners with the measure they have helped develop.

Another consideration is to also measure personality traits and response bias in the context of this research. It is expected that these characteristics could influence participants’ or clients’ responses and self-assessment. This is highlighted by previous research looking at individuals’ re-occurring patterns in relationships and the importance of having insight, managing expectations, and being open to collaborate with others [14, 15].

Alternative psychometric models could also be considered to continue understanding the existing scale. It could be that relationship sabotage is better understood as a dynamic system of interrelated behaviors—i.e., a psychometric network [79]—or a categorical latent variable—i.e., latent profile [80], as opposed to a latent variable framework. Although psychological constructs have traditionally been tested using a latent variable framework, and the scale in question showed excellent parameters using a CFA, it is important to continue exploring alternative ways to test and understand psychological phenomena.

Sample diversity could also be improved. Although the study recruited a culturally varied sample, the survey was only scored in English, which means that participants who do not speak English were no able to participate. Further, the sample was predominantly composed of female participants and answers from gender- and sexually-diverse individuals were minimal, which means specific conclusions are limited. Thus, it is a recommended step of scale development to test a newly developed scale with diverse samples and translated items [30], and it is expected that this step would provide further information towards making this scale more generalizable. Lastly the data was self-reported and cross-sectional. Thus, future studies should consider testing this instrument in clinical samples as well as using a design that includes “other’s” perceptions to better represent the couple dynamic or the other side of one’s intimate engagement.

### Clinical and theoretical implications

Understanding how self-sabotage is presented in romantic relationships has aided in the development of a scale from which practitioners can identify relationship issues and treat clients. The RSS is a brief scale that provides conclusive information about individual patterns in relationships. Findings using this scale can offer explanations regarding the reasons that individuals engage in destructive behaviours from one relationship to the next. Afterall, this measure is a product of consultations with practitioners working in the field of relationships. Also, the current project offers clear paths for future research to continue to engage in the validation of the scale and the development of models within the attachment and goal orientation frameworks to explain relationship dissolution and work towards relationship maintenance. Overall, this series of studies have filled the need to conceptualise and empirically measure relationship sabotage, and more broadly, it has complemented the literature on self-defeating attitudes and behaviours in relationships.

## Conclusion

The process of scale development requires a multi-study approach. Therefore, three studies were conducted. The first study was designed to pilot test the initial list of items using a EFA. The second study refined the scale and factor structure using a two-part EFA and one-congeneric model analyses. Lastly, the third study examined the final structure for the RSS with a CFA and reliability and construct validity analysis. The RSS was developed based on extensive investigations. The final result was a 12-item scale with three constructs (defensiveness, trust difficulty, and lack of relationship skills). Altogether, studies conducted thus far presented a new scale with reliable and valid dimensions (developed in a diverse and large sample) and robust evidence to build a model for predicting relationship sabotage and to inform future directions for relationships studies. Investigations should continue to test a model for sabotage in romantic relationships using the developed scale and other factors such as relationship differences and insecure attachment. More specifically, the relationship sabotage measure can be used to understand mediator constructs of relational outcomes within the attachment framework to explain relationship dissolution and work towards relationship maintenance.

## Data Availability

The Relationship Sabotage Scale manual with scoring and norming information can be requested from the corresponding author.
