# Correction to: The relationship sabotage scale: an evaluation of factor analyses and constructive validity

**DOI:** 10.1186/s40359-021-00661-z

**Published:** 2021-10-06

**Authors:** Raquel Peel, Nerina Caltabiano

**Affiliations:** 1 School of Psychology and Counselling, University of Southern Queensland, Building B; Room 340A, 11 Salisbury Road, Ipswich, QLD 4305 Australia; 2 Department of Psychology, James Cook University, 1/14-88 McGregor Road, Smithfield, Cairns, QLD 4878 Australia

## Correction to: BMC Psychol 9, 146 (2021) 10.1186/s40359-021-00644-0

Following publication of the original article [1], it was brought to our attention that, due to a processing error, links to references had been erroneously included in the first column of Table 2.

The erroneous links have since been removed from the table. The publisher apologizes for any inconvenience caused.

## References

[1] Peel R, Caltabiano N (2021). The relationship sabotage scale: an evaluation of factor analyses and constructive validity. BMC Psychol.

